# Advances in Xenotransplantation: Evaluation of αGal-KO Porcine Livers and Lungs Using Normothermic Machine Perfusion in a Collaborative Perfusion Hub

**DOI:** 10.3389/ti.2025.13781

**Published:** 2025-03-07

**Authors:** S. Stoerzer, S. Kruszona, P. Wand, H. Linge, H. Zlatev, K. Hoeffler, J. Singh, N. Roters, V. Muth, S. Tavil, A. Saipbaev, K. Cvitkovic, W. A. Kues, P. Zardo, F. Ius, J. Mengwasser, K. Splith, K. M. Schmidt-Ott, T. Goecke, R. Schwinzer, H. Niemann, A. Ruhparwar, M. Schmelzle, R. Ramm, P. Felgendreff

**Affiliations:** ^1^ Department of General, Visceral and Transplant Surgery, Hannover Medical School, Hannover, Germany; ^2^ Department for Cardiac, Thoracic, Transplantation and Vascular Surgery, Hannover Medical School, Hannover, Germany; ^3^ Lower Saxony Center for Biomedical Engineering, Implant Research and Development (NIFE), Hannover, Germany; ^4^ Biotechnology/Stem Cell Physiology, Institute of Farm Animal Genetics (FLI), Federal Research Institute for Animal Health, Neustadt, Germany; ^5^ Biomedical Research in End Stage and Obstructive Lung Disease Hannover (BREATH), German Center for Lung Research (DZL), Hannover, Germany; ^6^ Department of Nephrology and Hypertension, Hannover Medical School, Hannover, Germany; ^7^ Department of Gastroenterology, Hepatology, Infectious Diseases and Endocrinology, Hannover Medical School, Hannover, Germany

**Keywords:** xenotransplantation, liver, normothermic machine perfusion, genetically modified pigs, EVLP, perfusion hub, bridging therapy

## Abstract

Recently, initial clinical experience has been gained with the xenotransplantation of pig organs such as heart and kidney into terminally ill human patients in an effort to overcoming organ shortage. Here, we investigated the use of normothermic machine perfusion (NMP) to advance xenotransplantation research and develop bridging therapies for acute organ failure such as the use of pig livers as a liver dialysis system. We simultaneously analyzed livers and lungs from genetically modified pigs, carrying a knock-out of the GGTA1 gene, which is essential for xenoreactive αGal-KO-epitopes, by applying clinically established normothermic perfusion systems, solutions and human blood. Experiments involved perfusing organs with cell-free solutions as well as human erythrocyte concentrates for up to six hours, analyzing organ quality using invasive and non-invasive methods, and the isolation and analysis of immune cells from the perfusate. The results obtained show stable flow characteristics with physiological perfusion and oxygenation levels of the organs, and a largely intact organ architecture, confirmed by histological sections before and after perfusion. Overall, this study demonstrates the feasibility of normothermic machine perfusion of xenogeneic organs by an interdisciplinary team, thus paving the way for clinical applications of porcine xenografts involving NMP.

## Graphical Abstract

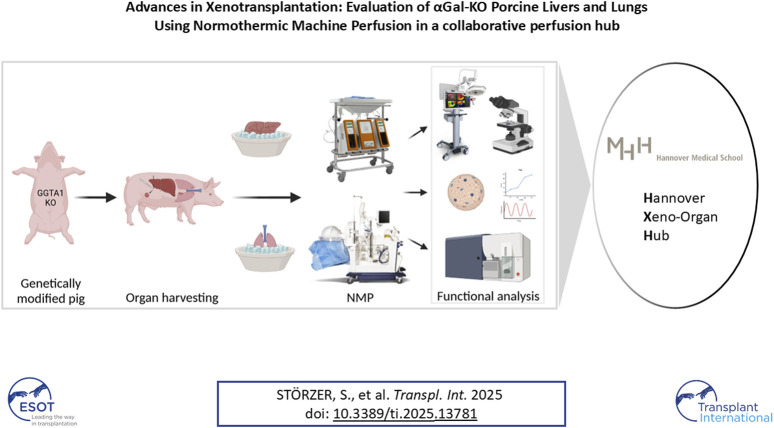

## Introduction

The critical shortage of donor organs poses a significant barrier to organ transplantation in patients with end-stage organ disease. Even using organs from aged and already diseased donors (extended criteria donors) the utilization rate for donor lungs in the US remains relatively low, at approximately 30% [1]. In addition, the increasing donor age, fibrosis and Metabolic dysfunction-associated steatohepatitis (MASH) in liver grafts results in diminished graft acceptance. Consequently, this trend in donor organ quality leads to extended waiting lists and unacceptably high mortality rates among patients awaiting a transplantation [2]. Therefore, new innovative sources for donor organs are needed to close the gap between available and required organs.

One promising approach to overcome critical organ shortage is the use of porcine organs for human transplantation approaches (xenotransplantation). Due to the high degree of anatomical and physiological similarity, porcine liver and lung represent a promising source for this experimental approach and have great potential for clinical application.

However, a direct transplantation of porcine grafts into humans is generally prohibited due to the immediate activation of the immune system or incompatibilities in the proteins that regulate complement formation or blood coagulation (reviewed in [3]).

Therefore, the decent aim of ongoing xenotransplantation research is to implement genetic modifications into the genome of donor pigs (reviewed in [4]) to enhance the compatibility of the porcine organs with the human immune system (reviewed in [5]).

Based on this approach, several highly specialized research centers have produced pigs with multiple knockouts and several different human transgenes. By implementing these modifications into pigs, special attention was paid on controlling the human complement system, to provide anticoagulation and ultimately to prevent death of the porcine tissue and cells (reviewed in [6]).

All these developments have already contributed to first promising results of xenotransplantation in preclinical and clinical settings. Especially by transplanting porcine hearts and kidneys in non-human primates (NHP) and first human patients, postoperative survival of 6–7 weeks were already reported [7, 8].

In contrast, xenotransplantation of porcine lung and liver has not yet reached these long survival periods. The first transplantations of these organs in NHP have demonstrated short-term organ survival rates in preclinical trials (reviewed in [9]).

To improve these first results and to extend the survival time following xenotransplantation, a pre-transplantation testing of genetically modified liver and lung is required. By using human blood components, the modified organs can be tested for functional, immunological and coagulation aspects of the grafts prior to the transplantation process.

Next to hypothermic perfusion systems [10] normothermic machine perfusion (NMP) has emerged as a very useful tool to evaluate and preserve organs prior to transplantation [11]. Both, the Liver Assist™ (XVIVO) system and the XPS system with multiple options of adapting the physical perfusion settings (perfusion or ventilation pressure, perfusion temperature) and improved analysis options, provide nearly optimal conditions for translating the xenotransplantation approach into the clinic.

However, the development of genetically modified animals as well as the functional evaluation of the grafts prior to the transplantation process requires a high level of expertise that can only be provided by a centralized xenotransplantation hub.

To support the establishment of such a center for xenotransplantation, we evaluated the challenges associated with xenoperfusion using human blood in a preclinical setting. Using pigs genetically engineered to lack αGal expression together with clinically certified Liver Assist™ (XVIVO) and XPS™ (XVIVO) systems, viability of explanted porcine grafts in such a centralized hub have been as assessed.

## Materials and Methods

### Study Design

Extracorporeal liver and lung perfusion was performed with organs from four genetically modified pigs. To enable the perfusion, pigs lacking the major xenoantigen αGal (αGal-KO), that were created by knocking out the GGTA1 gene were used for this study [12]. Animals deficient for GGTA1 were generated by breeding of parental lines carrying a homozygous or heterozygous knockout of the GGTA1 gene. Offspring was tested by PCR using saliva or tissue biopsy. The knockout was confirmed on porcine peripheral blood mononuclear cells (PBMCs) by flow cytometry analysis prior to organ procurement. Organ procurement was performed according to German animal welfare guidelines using a non-heart-beating donor model at the Friedrich-Loeffler Institute of Farm Animal Genetics in Mariensee (Neustadt). During the procedure, liver and lung were perfused with PERFADEX^®^ Plus solution containing 2,000 I.U. Heparin in preparation for subsequent extracorporeal machine perfusion. For perfusion, the organs were transported on ice to the surgical research lab, at Hannover Medical School (MHH). The extracorporeal machine perfusion of lung and liver was performed on XVIVO XPS System (Serial number: XPS0132), and the XVIVO Liver Assist™ for up to 6 h. Tissue and perfusate samples were taken to monitor machine perfusion and organ-specific functions on a regular basis. Additionally, hyperspectral images (HSI) were captured prior to and during perfusion in the respective organs. Perfusion success, defined as maintained organ perfusion over time, was evaluated in consideration of the respective tissue, perfusate, bile and HSI data.

### Surgical Procedures

Without premedication, animals were electrically stunned and killed according to standard procedure. After animals were exsanguinated, the abdominal cavity was opened immediately and the abdominal aorta and vena cava were identified.

Following cannulation of both vessels, perfusion of the organs was started with 2 L of ice-cold PERFADEX^®^ Plus solution containing 2,000 I.U. Heparin. Simultaneously, the abdominal aorta was ligated shortly behind passing through the diaphragm. During *in situ* perfusion, a sternotomy was performed, and lung and liver were explanted according to the German guidelines of organ procurement [13]. Following explantation, both organs were perfused again at the side of retrieval with 2 L of ice-cold PERFADEX^®^ Plus containing 2,000 I.U. Heparin (lungs) or with Histidine-tryptophan-ketoglutarate/Custadiol^®^ (HTK, plus 2,000 I.U. Heparin) (liver) in preparation for extracorporeal organ perfusion.

### Extracorporeal Liver Perfusion Using the XVIVO Liver Assist™

Following liver procurement procedure and 2 h of transport period to the MHH at +4°C, the explanted liver was prepared for extracorporeal organ perfusion. Back table, the portal vein and hepatic artery were cannulated using a 24 Fr portal vain and 3,33 Fr hepatic artery cannula. A representative liver tissue sample from the median liver lobe was taken for subsequent histological analysis. Furthermore, the common bile duct was drained by inserting a 10 CH suction tube (Asid Bonz, Herrenberg, Germany). Simultaneously, the XVIVO Liver Assist™ was primed with two bags of human erythrocytes of type 0 Rh^+^ and Gelafundin to reach a total perfusion volume of 2 L. Heparin (25,000 I.U.), Ilomedin (20 μg/mL (1:10 dilution); 2 mL/h), Insulin (1 mL/h minimum), 2 L/min of oxygen and 0.5–1 L/min of CO_2_ were added continuously for maintaining physiological perfusion conditions. The graft was connected to the XVIVO Liver Assist™ to perfuse the liver under physiological conditions with a portal vain flow of 550 mL to 450 mL/h and an arterial flow of 0.1–0.2 L/min for up to six hours. Hourly blood gas analysis was performed throughout the perfusion period to monitor the composition of the perfusion solution.

### *Ex Vivo* Lung Perfusion (EVLP)

After approximately two hours of cold ischemia during transport to the MHH, EVLP was performed according to the Toronto protocol [14]. The system was first primed with 1.5 L of STEEN Solution™ (XVIVO Perfusion AB, Moelndal, Sweden) and a total of 10,000 I.U. of heparin. The flow rate was set at approximately 40% of the equivalent cardiac output and the temperature was set at 34°C. Next to priming the device, the lung was prepared for the perfusion by inserting a endotracheal tube with 9 mm inner diameter (Ruesch, Rommelshausen, Germany), and usage of the XVIVO Lung Canulla Set™ for the pulmonary artery and right atrium. A standard retrograde flush with 1 L PERFADEX^®^ Plus was performed in the grafts prior to connecting the organ to the EVLP. Then, recruitment was performed at least one time per run, with positive end expiratory pressure (PEEP) continuously increased to approximately 10 and maintained at 100% FIO_2_ for a total of 10 min. Within the first run, recruitment was conducted for longer times. EVLP runs were proceeded from 60 up to 160 min and a maximum of 1 L STEEN Solution™ was added during procedure.

In two EVLP runs (2nd & 4th), additional simulation of extracorporeal perfusion using human blood was achieved by using one bag of human erythrocytes of type 0 Rh+ added to the circulation at the end of the respective perfusion time. This modified perfusion setting was conducted for up to 15 min.

### Perfusion Monitoring During Machine Perfusion

During extracorporeal perfusion, the physical perfusion parameters were monitored continuously for both organs. For liver graft perfusion, additional samples of the perfusate were taken prior to perfusion and in two-hour intervals to monitor the metabolic function of the grafts (urea, creatinine, AST, ALT, GLDH, alkaline phosphatase, gamma-GT, total bilirubin, and ammonia). The sampling was conducted in our clinical laboratory. Additionally, a complete blood count was conducted prior to perfusion and again at 3 h and 6 h after initiating the perfusion to monitor any hematological changes.

### Hyperspectral Imaging During Liver and Lung Perfusion

HSI, using TIVITA^®^ 2.0 (TIVITA^®^ Tissue System, Diaspective Vision GmbH, Am Salzhaff, Germany), was conducted hourly during the perfusion. In addition, HSI was used during one exemplary run of EVLP prior to the perfusion and after each recruitment during perfusion STEEN Solution™ as well as alongside with the hemoperfusion.

The parameters hemoglobin oxygen saturation (StO_2_; 1 mm deep, 500–600 nm), tissue hemoglobin index (THI, 500–600 nm), near-infrared (NIR; 4–6 mm, 700–1,000 nm) perfusion index and tissue water index (TWI, 900–980 nm) were recorded.

### Assessment of αGal Epitopes in Porcine PBMCs

PBMCs were isolated from the blood of αGal-KO pigs, which were used as donors for perfusion experiments. PBMCs from a wildtype (wt) pig served as control. αGal epitopes were detected by staining with FITC conjugated *Griffonia simplicifolia* isolectin B4 (IB4-FITC). Analysis was performed on a FACS Calibur flow cytometer (Becton Dickinson, San Jose, CA, United States) and data were processed using FCS Express 7 (*De Novo* software, Pasadena, CA, United States).

### Flow Cytometry Analysis

Flow cytometry was employed to analyze perfusate samples during liver NMP to detect and quantify the release of porcine immune cells into the perfusate. Specific porcine antibodies detecting CD45 (Clone K252.1E4, Acris), CD3 (Clone BB23-8E6-8C8, BDBiosciences) CD21 (Clone LT21, Origene), CD4 (Clone 74-12-4, Acris), CD8 (Clone 76-2-11, Acris), CD14 (Clone MIL2, Serotec) and CD56 (Clone MEM-188, Biolegend) were used, allowing for identification and characterization of cellular components in the perfusate. For flow cytometry analysis, we used the BD FACSCalibur with CellQuest™ Pro software and FCS Express 7.

### Histology

Histological examination was performed in both organs, on different time points. During lung perfusion, tissue samples (2 cm × 2 cm) of each lobe were taken before and at the end of the perfusion interval. From liver grafts, samples (2 cm × 2 cm) were taken prior to connecting the organ to the extracorporeal system as well as at the end of the perfusion interval. All histological samples were fixed in 10% methanol buffered formalin for 24 h at room temperature prior to further processing.

Dehydration using increasing concentrations of ethanol and embedding in Paraffin was performed according to standard procedure. Sections of 2 µm thickness were cut using a microtome and stained applying a standard Hematoxylin and Eosin (H&E) protocol.

### Data Export and Statistical Analysis

Data export from the XVIVO XPS and Liver Assist™ systems was performed after each run. Perfusion data were then manually organized and processed. Statistical analysis was performed using appropriate methods for data validation and analysis to ensure reproducibility and significance of results. EVLP-Data were analyzed using R version 4.2.3 (2023-03-15), utilizing the ggpubr package to examine pulmonary arterial systolic pressure and left atrium oxygen partial pressure over the time during EVLP. Continuous variables were summarized using median values with interquartile ranges [IQR].

## Results

### General Animal Data

**FIGURE 1:**
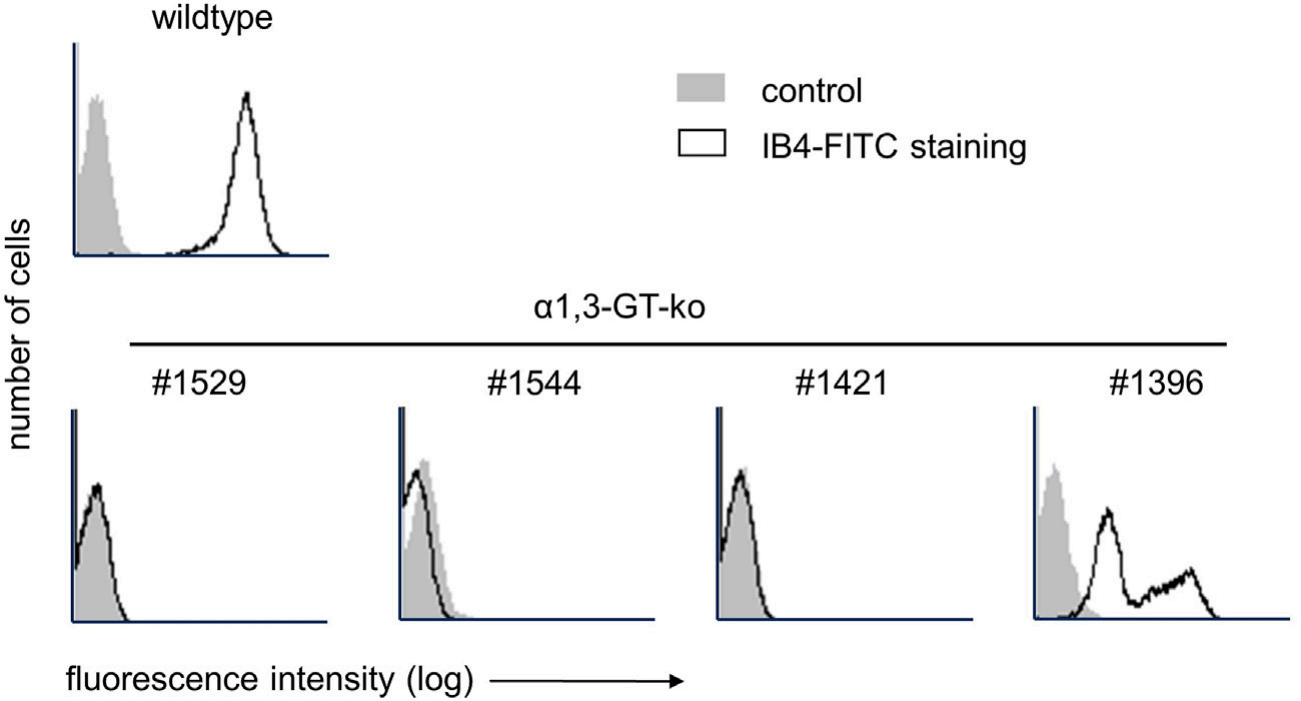
Flow cytometry analysis of αGal expression on PBMCs of genetically modified pigs with IB4-FITC. In the top row, staining on PBMC of a wild type pig is shown. The bottom row shows αGal expression on the four genetically modified pigs used for liver and lung machine perfusion.

In total, four female genetically modified pigs (chronological order of experiments #1529, #1544, #1421, #1396), and aged between 20 and 45 months, with a weight range of 250 kg–350 kg, were used for the extracorporeal organ perfusion experiments. Prior to organ procurement, the flow cytometry analysis of peripheral blood mononuclear cells (PBMCs) verified the absence of αGal epitopes, in three of four animals. Despite a confirmed bi-allelic knockout of the GGTA1-gene (deletions of one base (Δ1) and five bases (Δ5) within the coding from of GGTA1), PBMCs of pig #1396 stained positive for αGal (Figure 1). Further genetic analysis revealed a third, most likely functional, copy of the GGTA1-gene (not shown, probably reflecting a copy number variation for this locus). Due to the size of the animals, the liver weight was between 2,800 and 3,200 g, weight of the lungs was not determined. No evidence of intrahepatic clotting or pulmonary edema prior to the perfusion was observed in any of the organs.

### Extracorporeal Liver Perfusion Data

**FIGURE 2:**
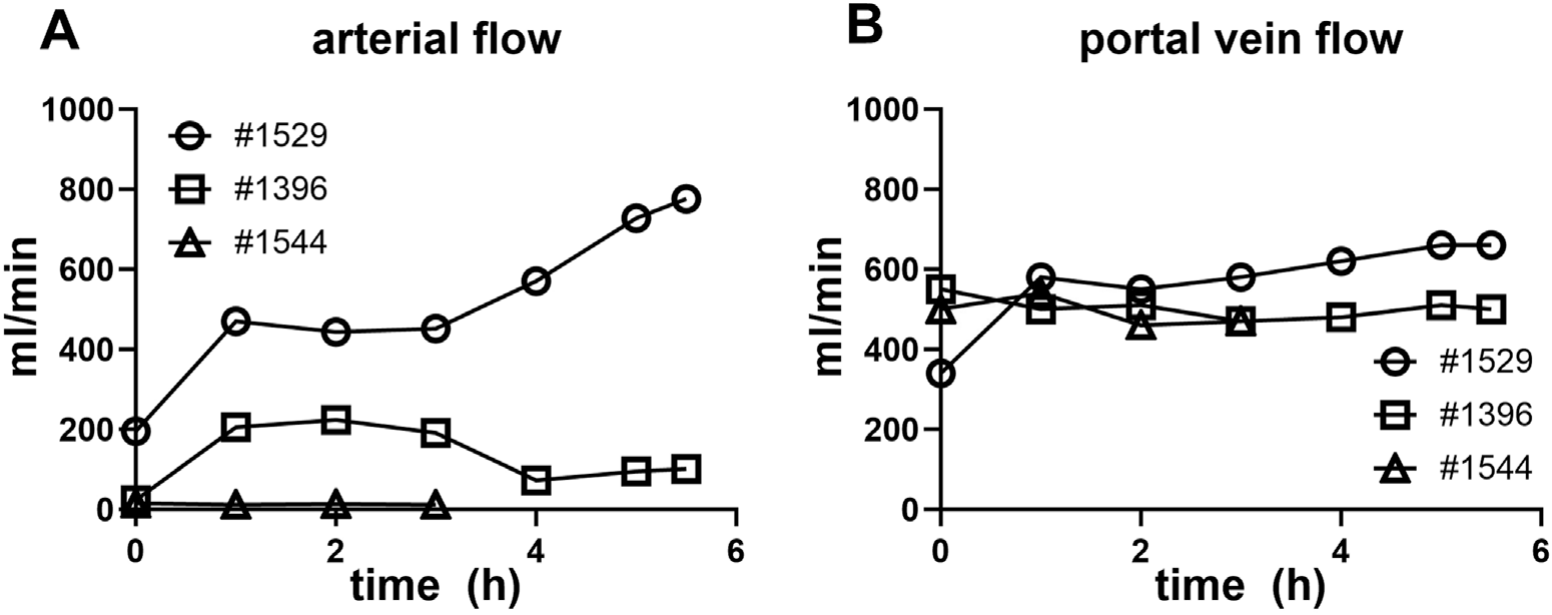
Liver NMP perfusate flow characteristics. **(A)** Arterial vein flow in mL/min over the duration of the NMP (conclusion at 3–6 h). **(B)** Portal vein flow in mL/min is shown over the duration of the normothermic liver perfusion (conclusion at 3–6 h).

Extracorporeal liver perfusion could be maintained for up to 252.5 ± 86.7 min. The extracorporeal liver perfusion was conducted under physiological conditions, with the following portal venous and hepatic artery flows: #1529, arterial flow 483.3 ± 169.4 mL/min, portal vein flow 0.518 ± 0.178 L/min, #1396, arterial flow 130.4 ± 70.4 mL/min, portal vein flow 0.503 ± 0.024 L/min, #1544, arterial flow 13.3 ± 1.3 mL/min, portal vein flow 0.492 ± 0.031 L/min (Figures 2A, B). The vascular resistance of liver #1544 in the portal venous system branches was already high at the beginning of perfusion (VR of 4.65 vs. 0.3 in liver #1529) and increased by more than 25% in the first hour of liver perfusion. In consideration of the decreasing hemoglobin and hematocrit values found in the blood gas analysis, perfusion of this particular organ was terminated after 3 h. The liver of #1421 was discarded, due to initial perfusion problems during graft procurement after DCD.

### Clinical Chemistry Data of the Perfused Livers

**FIGURE 3:**
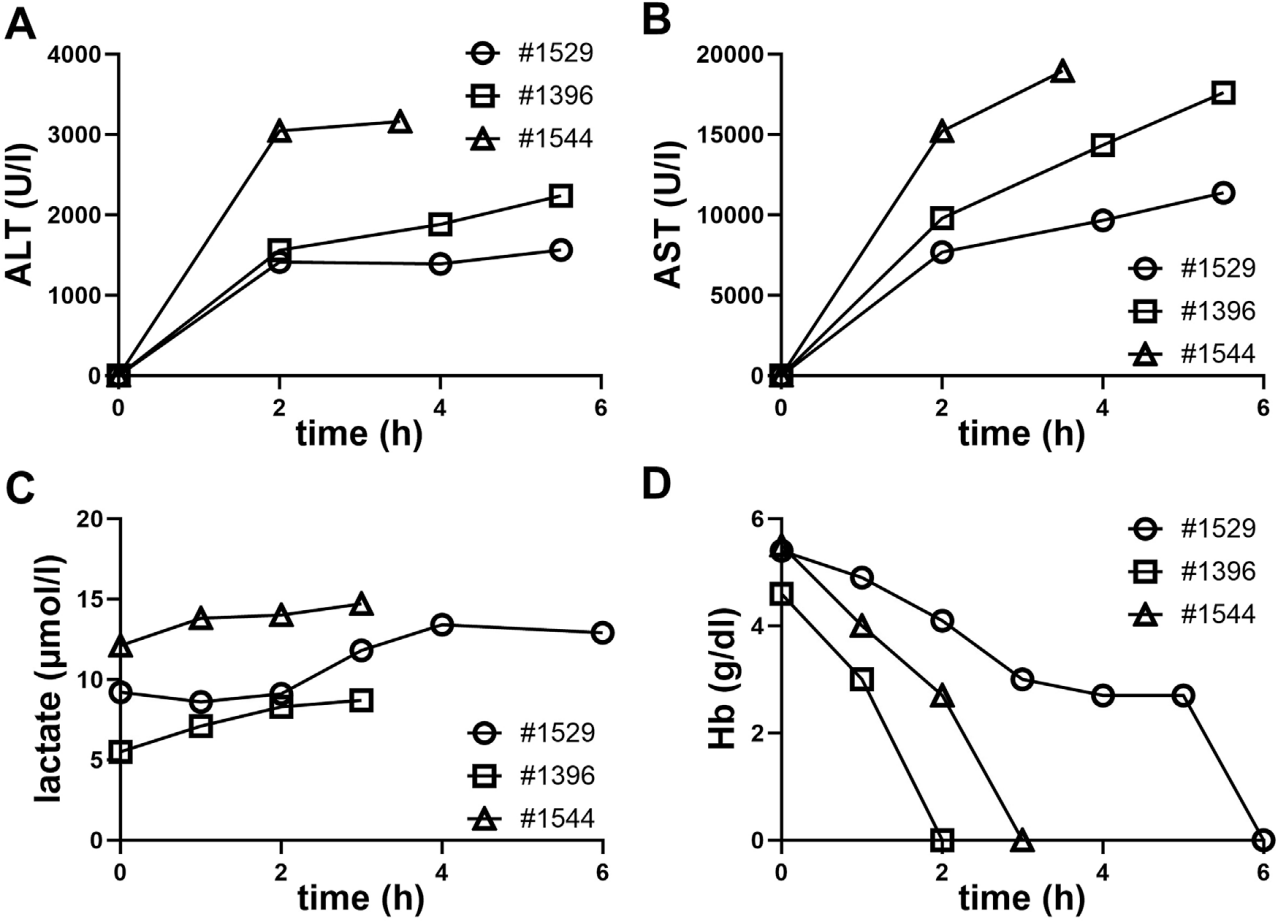
Clinical chemistry from NMP perfusates during the perfusion period. **(A)** Alanine aminotransferase (ALT), **(B)** aspartate aminotransferase (AST), **(C)** lactate, **(D)** hemoglobin (Hb).

Analysis of ALT and AST levels in three pigs over time during perfusion was performed (Figures 3A, B). ALT and AST levels increased over time in all three pigs, with pig #1544 showing the highest ALT and AST levels. The slope for pig #1544 is the steepest, indicating a faster increase in ALT and AST levels compared to the other pigs. ALT and AST levels increases during perfusion suggest liver stress or damage over time in all three pigs.

Also, a continuous increase in lactate levels was observed by blood gas analysis in all three perfusions. This rise in lactate suggests a shift towards anaerobic metabolism. Possible causes include hypoxia, insufficient perfusion into capillary structures or cellular stress or damage (Figure 3C).

The decline in hemoglobin levels, which were below the measurable threshold at termination of all three perfusions, is indicative of a substantial loss of erythrocytes.

Accelerated hemolysis could be due to mechanical stress of erythrocytes passing the perfusion pump or through contact with non-biocompatible surfaces within the perfusion apparatus. Additionally, the presence of porcine immune cells or the lack of human plasma in the perfusate could also lead to increased hemolysis.

The accompanying figure (Figure 3D), clearly shows a steady decline, ultimately reaching unmeasurable hemoglobin levels during perfusion. All measured clinical parameters underscore the physiological challenges encountered during the perfusion process.

### EVLP Perfusion Data

**FIGURE 4:**
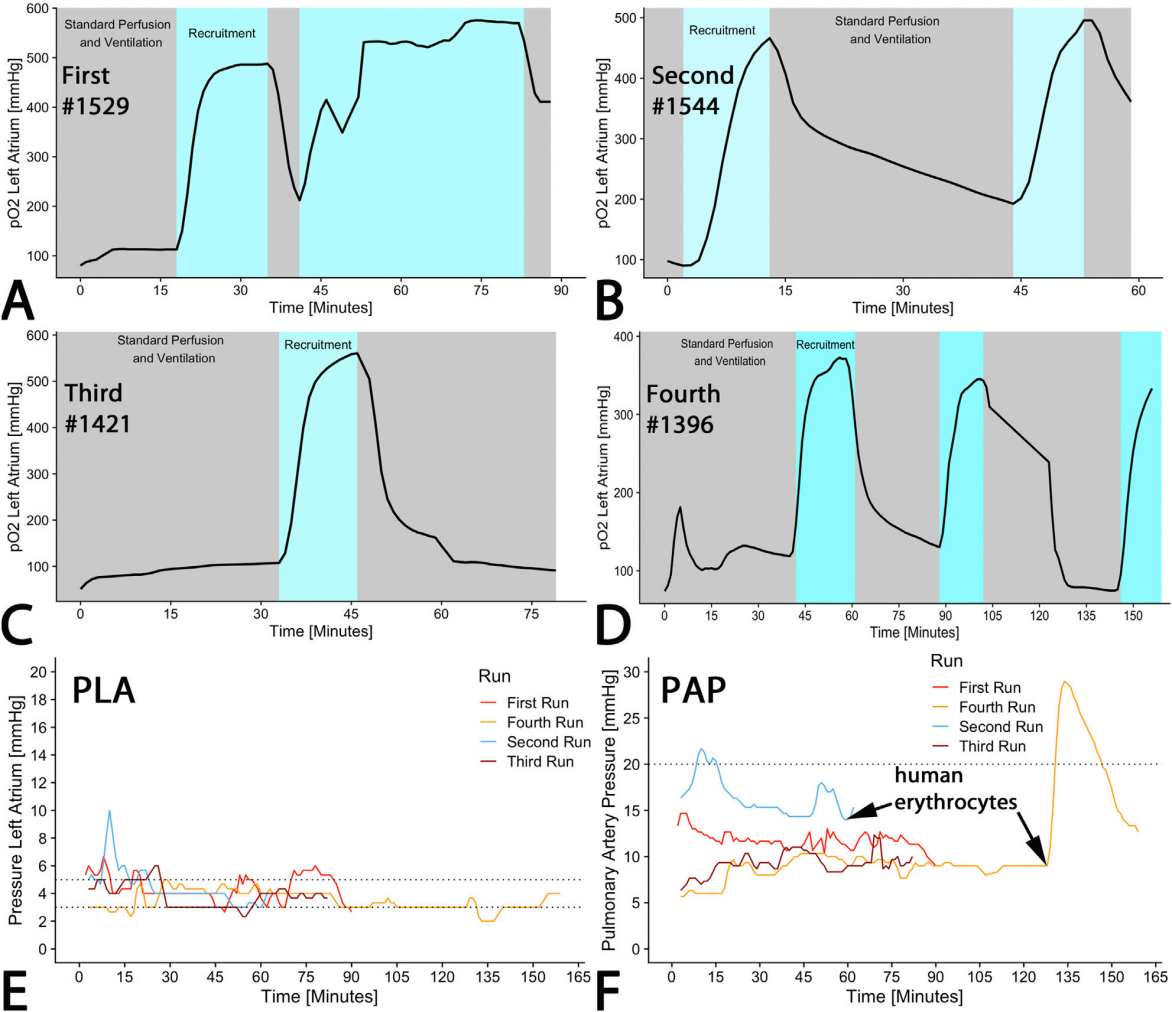
EVLP perfusion parameter. **(A–D)** Oxygenation of the perfusate was measured in the left atrium showing increase due to recruitment applying 100% oxygen as ventilation gas. **(E)** A recommended pressure of 5 mmHg at the outflow from the left atrium (PLA) was targeted. **(F)** Inflow pressure at the pulmonary artery (PAP) was measured, exhibiting a dramatic increase upon addition of human erythrocytes. The curves represent online measurements of each run.

Extracorporeal lung perfusion was maintained with STEEN Solution™ in all organs as planned for up to two hours. A significant increase of oxygen saturation in the left atrium (pO_2_(LA)) was observed in each run during recruitment maneuvers (100% FiO_2_) (Figures 4A–D). However, the pO_2_(LA) returned to its initial level following these maneuvers and did not improve further after repeated recruitments (Figures 4A–D).

According to the Toronto protocol, left atrium pressure (PLA) was intended to be between 3 and 5 mmHg. Although there were some brief periods in runs 1–3 where the pressure exceeded 5 mmHg, a median left atrium pressure of 4 mmHg was maintained across all runs (first run: 4 (4–5); second run: 4 (4–5), third run: 4 (3–4; fourth run: 4 (4–5); p < 0.001) (Figure 4E).

During regular perfusion with STEEN Solution™, pulmonary arterial pressure (PAP) should remain below a cut-off of 20 mmHg. With the exception of the initial phase of the first run and after erythrocyte administration in the fourth run, pressure was maintained within the targeted range (Figure 4F). The median PAP across all runs was 10 (9–14) mmHg (first run: 12 (11–12); second run: 16 (15–17), third run: 9 (8–10; fourth run: 9 (9–10); p < 0.001) (Figure 4F). The addition of human erythrocytes in run 4 (pig #1396) caused an increase in PAP to 30 mmHg, which subsequently declined (Figure 4F). In congruence with PAP, pulmonary vascular resistance (PVR) increased from 100 to 1,500 dyn/s/cm^−5^ within 5 min (Data not shown) immediately after addition of one erythrocyte concentrate at the end of run 4 (Figure 4F). Simultaneously, the Horowitz-index decreased from 400 to 160 mmHg within 3 min, and remained steady over 15 min and subsequently increased to 300 mmHg (not shown). Within 15 min after reaching peak values, PAP and PVR decreased to normal levels.

### Flow Cytometry

**FIGURE 5:**
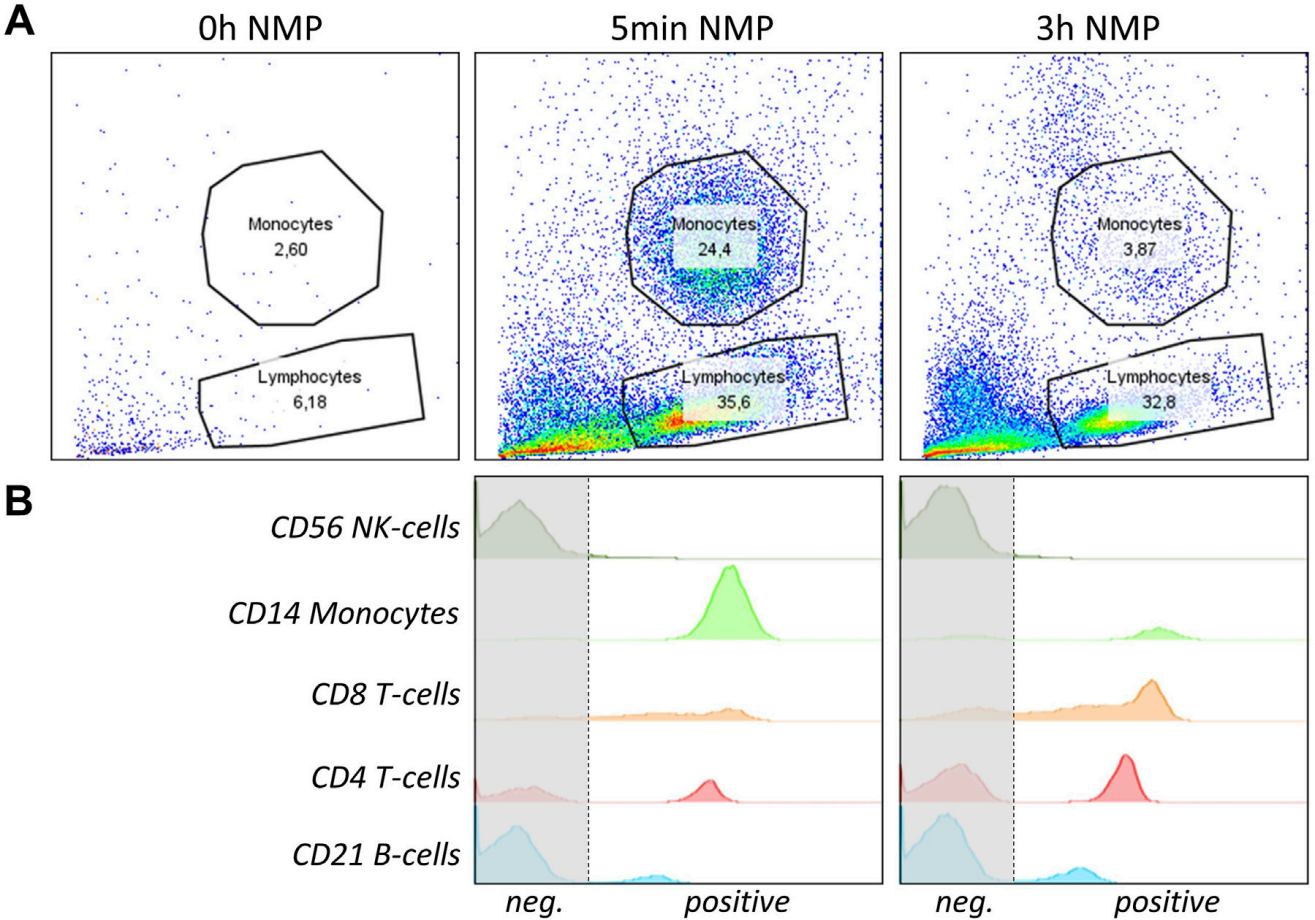
NMP perfusate immune cell analysis of a representative pig liver is shown. **(A)** FSC, SSC dot blots with lymphocyte and monocyte gating of liver perfusate before perfusion (0 h), at 5 min and 3 h into perfusion are shown. **(B)** Flow cytometry histograms of specific immune cell populations isolated from the perfusate of NMP after 5 min and 3 h of perfusion are shown. Cells were gated to be lymphocytes or monocytes and to be CD45^+^. From top to bottom, anti-human CD56 (NK-cells), CD14 (Monocytes), CD8 (cytotoxic T-cells), CD4 (T-helper cells) and CD21 (B-cells) were used to identify specific populations. Graphs represent single measurements.

Flow cytometry analysis demonstrated the presence of specific porcine immune cell populations, including NK cells (CD56), monocytes (CD14), T cells (CD4/CD8), and B cells (CD21)) in the perfusate during perfusion. FSC, SSC dot blots show no immune cells in the perfusate before perfusion, but significant amounts at 5 min and 3 h into perfusion (Figure 5A). The analysis indicated an enrichment of B and T cells and a decrease in monocytes after 3 h NMP compared to the 5 min time point (Figure 5B). Only few NK cells were detectable at both time points with no significant difference in NK cell numbers between the 5 min and 3 h time point (Figure 5B). The persistence of immune cells in the perfusate until late into the NMP suggests active immune cell release from the liver graft during the perfusion period.

### Hyperspectral Imaging

**FIGURE 6:**
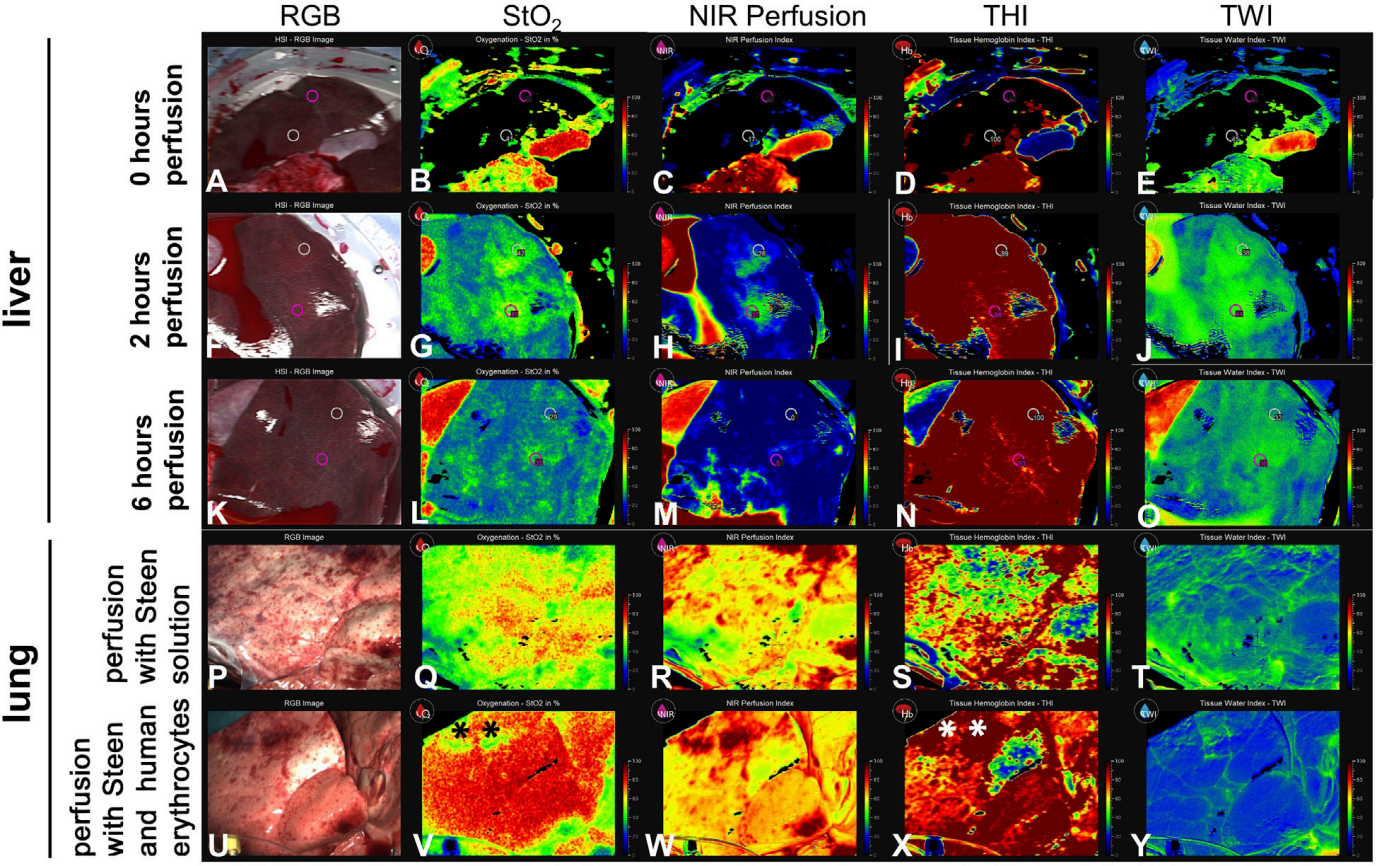
Series of images showing hyperspectral imaging of liver and lung during NMP. **(A–O)** The images illustrate different parameters of liver tissue, including **(B, G, L)** tissue oxygen saturation (StO_2_), **(C, H, M)** NIR Perfusion Index, **(D, I, N)** Tissue Hemoglobin Index (THI), **(E, J, O)** Tissue Water Index (TWI) at 0 h, 2 h, 6 h of NMP. Measurements in livers were taken in a central and a peripheral area (white and pink circles) **(P–T)** Lung perfused with cell-free STEEN Solution**™**. **(U–Y)** Lung perfused with STEEN Solution**™** containing human erythrocytes, directly after recruitment. (*) Area of venous congestion. Values for StO_2_, NIR perfusion index, THI, TWI are arbitrary units.

HSI provided additional insights into the tissue condition during perfusion (Figures 6A–Y). The NIR index started at a baseline value indicating undisturbed perfusion of the liver. During the first 2 h, there was a noticeable increase in NIR values (Figure 6H). From the third hour onwards, NIR values decreased and plateaued at a low level at the end of the 6 h perfusion (Figure 6M). Initial THI measurements suggested a normal distribution of hemoglobin within the liver tissue. Over the 6-h period, the THI values remained within a consistent range, showing no signs of significant hemoglobin depletion or concentration (Figures 6D, I, N). Perfusion heat maps displayed uniform perfusion across superficial liver tissue, with high perfusion areas correlating with high oxygenation levels. When comparing StO_2_ values at the start of the perfusion (0 h) with measurements at 2 h, an increase can be observed (0 h: central 41%/periphery 0%; 2 h: 49%/42%), at the peripheral measurement point. After 6 h, we observed decreased StO_2_ values (35%/29%). NIR values at 2 h (37/26) versus 6 h NMP (5/0) also show a significant decrease. The same decrease is seen for TWI (2 h: 44/38 vs. 6 h: 36/32), but not for THI (2 h: 98/99 vs. 6 h: 93/100).

During EVLP, substantial differences caused by the addition of human erythrocytes to the cell-free STEEN Solution have been observed applying HSI. After addition of the erythrocyte concentrate, oxygenation and THI increased compared to only STEEN Solution™ (Figures 6Q, S, V, X). In contrast, TWI decreased in most areas (Figures 6T, Y). Area of venous congestion (*) could be identified exhibiting low StO_2_ and high THI in lungs perfused with erythrocytes (Figures 6V, X).

### Histological Results

**FIGURE 7:**
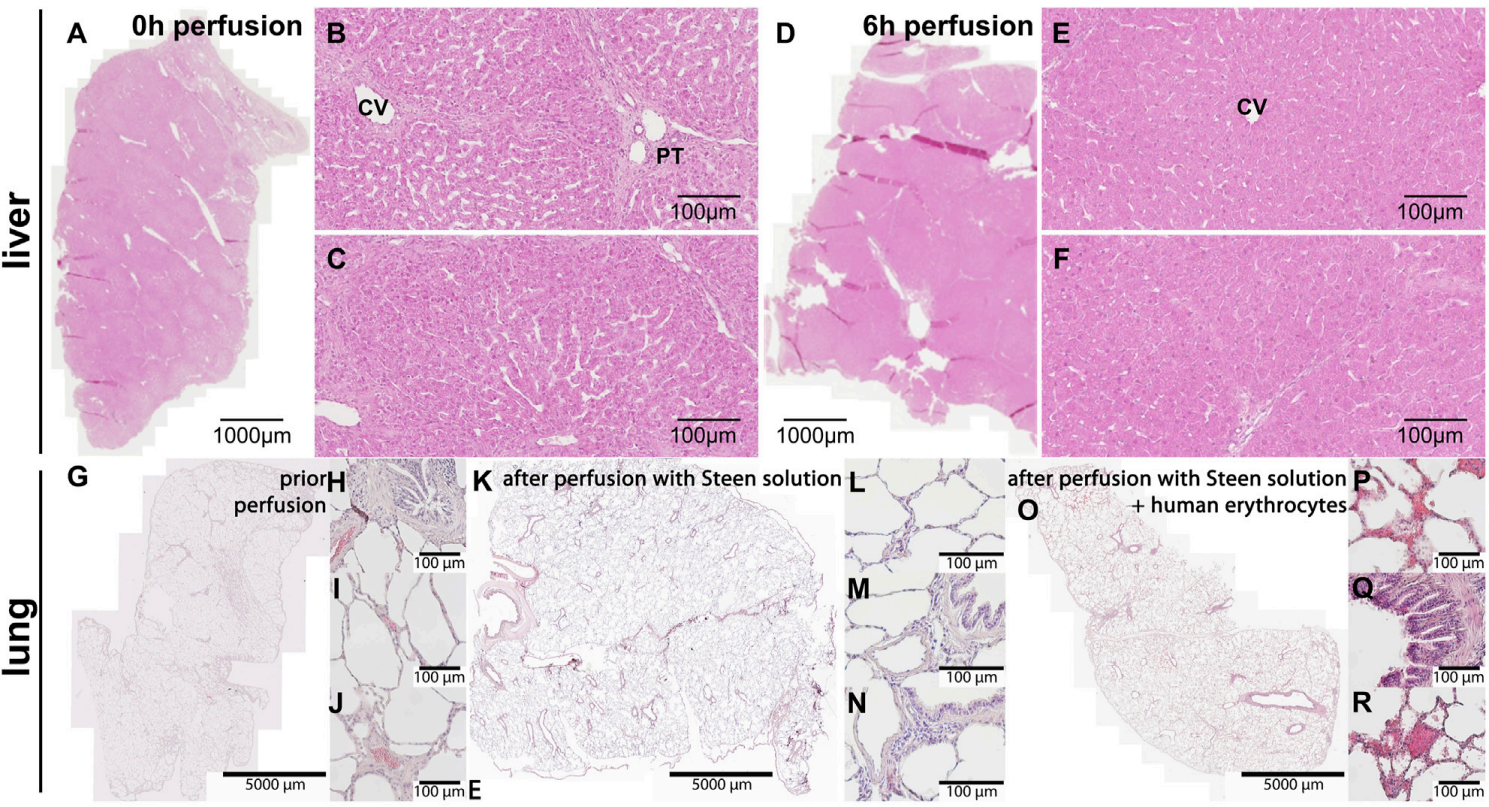
Hematoxylin & Eosin staining of the liver and lung tissue samples. **(A–C)** Liver and **(G–J)** lung before the start of perfusion. **(D–F)** Liver and **(K-N)** lung at the end of perfusion. **(B, C)** At 0 h intact hepatic parenchyma with clearly visible sinusoids around central vein and a portal triad is visible. **(E, F)** At 6 h perfusion hepatocytes still look intact, but sinusoidal space has markedly collapsed. CV central vein, PT portal triad. **(G–J)** The images show intact lung histoarchitecture without edema formation in sections directly after retrieval and **(K–N)** after 2 h of cell-free perfusion with STEEN Solution^**TM**^. **(H–J)** Porcine erythrocytes were visible within small vessels and capillaries directly after retrieval, **(L–N)** but were absent after 2 h of cell-free perfusion. **(O–R)** Following perfusion with human erythrocytes, erythrocytes were found within alveoli and bronchial epithelium. Scale bars represent **(A, D)** 1,000 μm, **(G, K, O)** 5,000 µm or 100 µm.

The histological examination of peripheral liver tissue samples taken prior to perfusion (Figures 7A–C) and at the end of perfusion (Figures 7D–F), revealed shrunken periportal fields and partly collapsed sinusoidal space at 6 h perfusion. No significant necrotic areas were detected. Nuclear staining remained nearly unchanged as most cell nuclei were well-defined (Figures 7B, C, E, F). The imperfect perfusion described above, did not seem to cause extensive cell death or tissue damage in the liver.

Lung tissue exhibited intact lung architecture before and after perfusion with cell-free STEEN Solution™ (Figures 7G–N). Compared to lungs directly after harvesting, vessels and capillaries were free of erythrocytes (Figures 7K–N). Edema formation could not be observed. After addition of human erythrocytes, extravasation of erythrocytes into the airspace, (alveoli as well as bronchi) could be observed, indicating a loss of barrier function (Figures 7O–R).

## Discussion

This study presents initial results of our extracorporeal machine perfusion of lungs and livers isolated from αGal-KO pigs, demonstrating the feasibility of xenogeneic perfusion using clinically approved devices in a realistic setting. In combination with the also established first structures of a xenotransplantation hub including administration and analytic pathways. All these achievements will be the first steps towards building an interdisciplinary xenoperfusion hub, for implementing xenotransplantation in a clinical application.

### Extracorporeal Liver Perfusion

In this study, we utilized genetically modified pigs that do not express αGal, aiming to reduce immunogenicity, a key target in xenotransplantation [15]. This approach of genetic modification, particularly the knockout of xenoantigens such as αGal, Neu5Gc and Sda, alongside the introduction of immunoprotective human transgenes, is well documented in the literature. For instance, Detelich et al. noted maximum liver perfusion times of 5–7 h in wild-type pigs, 4–6 h in αGal-KO/hCD55 pigs, and 8–14 h in pigs with multiple knockouts and transgenes. Here, we achieved satisfactory perfusion parameters in 3 out of 4 normothermic liver perfusions. We stopped perfusion at 6 h at the latest, but we achieved comparable perfusion times as in the αGal-KO/hCD55 group reported by Detelich et al. [15, 16].

Histological analysis revealed intact tissue, lacking major necrotic areas and well-defined hepatocyte nuclei after 6 h of perfusion, indicating that the liver’s structural integrity had been preserved. These encouraging results suggests that the present NMP method is capable of maintaining liver structures over extended periods.

Hyperspectral imaging demonstrated consistent perfusion during the initial hours. Despite a constant THI at the end of the perfusion period, StO_2_, NIR and TWI decreased, indicating hypoxia or ischemia. A drop in StO_2_ values has also been observed in other studies, often attributed to reduced oxygen delivery or increased oxygen consumption [17, 18].

The question of how long a liver can be safely perfused prior to irreversible damage needs to be addressed in future experiments. Therefore, further developments should involve optimizing the perfusion parameters to ensure consistent oxygen delivery throughout the perfusion period. Additionally, exploring other genetically modified pig lines could yield better outcomes.

Another significant observation was the release of porcine immune cells into the perfusate, including NK cells (CD56), monocytes (CD14), T cells (CD4/CD8), and B cells (CD21). This phenomenon of passenger leukocytes is also observed in lung perfusion, leading to the implementation of leukocyte filters in clinically used perfusion circuits [19]. Understanding the nature and impact of these immune cells is crucial for developing strategies to mitigate any adverse effects [20, 21].

### *Ex Vivo* Lung Perfusion

The lung is particularly challenging for xenotransplantation, because there is no preclinical or clinical model demonstrating long-term survival after xenotransplantation. Even human lung transplantation remains suboptimal compared to other organs, with a median survival of approximately 7 years according to ISHLT data [22].

Here, by applying Steen solution we have successfully demonstrated reproducible cell-free perfusion of genetically modified porcine donor lungs exhibiting excellent functionality. This method forms the basis for our future preclinical research to enable xenogeneic lung transplantation. In pig-to-non-human primate (NHP) lung transplantations most lungs failed to function and showed rapid loss of barrier function and extravasation of human erythrocytes [23]. Although, tested only as an example and without functional human immune cells, platelets, antibodies or complement, PAP, PVR and extravasation of human erythrocytes increased after addition of human erythrocyte concentrates. In our opinion, specific interactions between porcine lungs and human erythrocytes are responsible for the extravasation of human erythrocytes, even though some damage to the endothelium will occur in any organ perfusion. Some mechanisms have been reported, such as binding of human erythrocytes by porcine macrophages using sialoadhesin [24]. We speculate that there may be other, as of today unknown, mechanisms or molecular incompatibilities responsible for these observations.

In general, the use of human erythrocyte concentrates in the perfusate instead of human whole blood, is the major limitation of this study. The absence of functional human complement, antibodies and immune cells might explain the good perfusion results achieved with organ from pig #1396 that appeared to express αGal in certain amounts on PBMCs, possibly through chromosomal recombination.

Our results show that the use of porcine DCD organs without premedication of the animal is feasible in this context, especially for lungs confirming previous experiments using wildtype pigs [25, 26]. Tissue integrity and functional recovery of retrieved organs could improve by further reducing cold and warm ischemia times and donor animal anti-coagulation [27].

## Conclusion and Outlook

Here, we demonstrate that normothermic machine perfusion of genetically modified porcine lungs and livers using human erythrocyte concentrates for up to six hours is feasible in an interdisciplinary setting. Despite facing challenges due to the use of DCD organs and the absence of certain human blood components in the perfusion medium, our results are encouraging, especially regarding the establishment of a xenotransplantation hub. Using clinically approved solutions and perfusion devices, physiological perfusion conditions, confirmed by hyperspectral imaging and blood gas analysis.

The results of this study, including the successful perfusion of genetically modified porcine organs, representing first steps in the development of the Hannover Xenotransplantation Hub. The development of this hub is essential for focusing the knowledge of genetic modeling as well as functional assessment of porcine organs in order to transfer this innovative concept into clinical practice. Taking this approach as well as the presented results into account, a variety of clinical applications from short-term to long-term organ replacement can be considered.

Our next step will be the development of a organ assist device as a treatment option for patients with acute organ failure. Especially for the liver, advanced NMP of porcine livers is becoming a treatment option for patients with acute liver failure. To achieve this aim, long-term studies using whole human blood as well as multi-knockout, multi-transgene animals will be performed. Furthermore, detailed studies on the immunological interactions between the porcine grafts and the human recipient’s need have to be performed in preparation for a subsequent organ transplantation.

Considering the establishment of specialized organ perfusion centers around the world, donor organ evaluation and conditioning is becoming popular and is reaching the level of a commercial service.

These centers benefit from centrally available equipment and high level of expertise in handling technology and offer a path towards organ repair [28]. Based on the comprehensive analysis and findings from our research, it is our strong opinion that the establishment and advancement of a dedicated perfusion hub is essential for future clinical application of xenotransplantation. The creation of such a hub would facilitate interdisciplinary collaboration, bringing together expertise from various fields including surgery, immunology, molecular genetics and bioengineering. This collaborative environment is crucial for addressing the complex challenges associated with xenogeneic organ perfusion and transplantation. The recently shown potential of NMP mediated knock-down of porcine MHC class-I (SLA-I) to avoid organ rejection [29] after allotransplantation might be promising also for porcine xenografts prior to clinical transplantation to terminally ill patient.

The concept of a perfusion hub not only allows for the pooling of resources and specialized equipment but also promotes the exchange of knowledge and best practices among experts. This set up would enable refinement of perfusion techniques, optimization of genetic modifications, and development of new protocols to improve the viability and functionality of donor organs. By centralizing these efforts, we could accelerate the translation of research into clinical practice, ultimately improving patient outcomes and addressing the critical shortage of donor organs.

In conclusion, the establishment of perfusion hubs, coupled with interdisciplinary collaboration, holds great promise for the future of xeno- and allotransplantation, providing new hope for patients in need.

## Data Availability

The raw data supporting the conclusions of this article will be made available by the authors, without undue reservation.
